# HAIRY POLYP on the dorsum of the tongue – detection and comprehension of its possible dinamics

**DOI:** 10.1186/1746-160X-8-19

**Published:** 2012-07-09

**Authors:** Edela Puricelli, Marinez Bizarro Barra, Bruno Hochhegger, Deise Ponzoni, Henrique Voltolini de Azambuja, Mário Alexandre Morganti, Jorge Henrique Schmitt

**Affiliations:** 1 Department of Oral and Maxillofacial Surgery, Hospital de Clínicas de Porto Alegre, (Ramiro Barcelos, 2350), Porto Alegre, RS, (900350-903), Brazil; 2 Department of Pathology, UCSPA, (Sarmento Leite St 245), Porto Alegre, RS, (90050-170), Brazil; 3 Department of Radiology, UCSPA, (Sarmento Leite St 245), Porto Alegre, RS, (90050-170), Brazil; 4 Department of Dentistry, School of Dentistry - UFRGS, (Ramiro Barcelos St, 2492), Porto Alegre, RS, (900035-003), Brazil; 5 Department of Anesthetist at Irmandade Santa Casa de Misericordia de Porto Alegre, (Professor Annes DiasSt, 295), Porto Alegre, RS, (90010-170), Brazil

**Keywords:** Hairy Polyp, Computed Tomography, Benign Tumor

## Abstract

**Background:**

The formation of a Hairy Polyp on the dorsum of the tongue is a rare condition that may hinder vital functions such as swallowing and breathing due to mechanical obstruction. The authors present the situation on a child with an approach of significant academic value.

**Methods:**

Imaging diagnostics with the application of a topical oral radiocontrastant was used to determine the extent of the tumor. Performed treatment was complete excision and diagnostics was confirmed with anatomopathological analysis.

**Results:**

The patient was controlled for five months and, showing no signs of relapse, was considered free from the lesion.

**Conclusion:**

Accurate diagnostics of such a lesion must be performed in depth so that proper surgical treatment may be performed. The imaging method proposed has permitted the visualization of the tumoral insertion and volume, as well as the comprehension of its threatening dynamics.

## Introduction

Although the Hairy Polyp, also called Teratoid Polyp, has its origins and classification hugely debated [1-3], authors do agree that it is a benign and rare lesion. It’s essentially composed of normal tissue located at an abnormal place, containing elements of the ectodermal and mesodermal germ layers [1,2,4,5]; such as adipose, glandular and muscular tissues, covered by epithelial squamous stratified tissue.

Hairy Polyp is more frequently seen among women, mainly newborns, scarcely happening to adults [2]. Its presence in the tongue isn’t common because, at such location and in considerable volume, it could undermine breathing and swallowing [2,3,5-9].

For diagnostic purposes, imaging exams such as computed tomography (CT) or magnetic resonance (MRI) might be performed. Particularly we indicated the computed tomography with a topic radiocontrast as it may not only detected the presence, but also the dynamics of a tumor located on the dorsum of the tongue. This paper, therefore, aims to describe and discuss the presence of a Hairy Polyp in the tongue of a newborn girl, rapidly developing into complications regarding the vital functions of swallowing and breathing.

## Case report

A 90 days old female patient, according to information given by the mother, had shown progressive signs of difficulty in swallowing and breathing, together with repeated incidences of cough and vomiting. The constant refusal to suck the pacifier and to be breastfed, meant that it took a long time to feed the baby. The medical report informed that there was a delay in weight gain. At the first medical examination, a voluminous and mobile tumoral alteration was noticed on the dorsum of the tongue, resting anteriorly, but it wasn’t possible to stipulate whether it extended to the oropharyngeal area.

**Figure 1:**
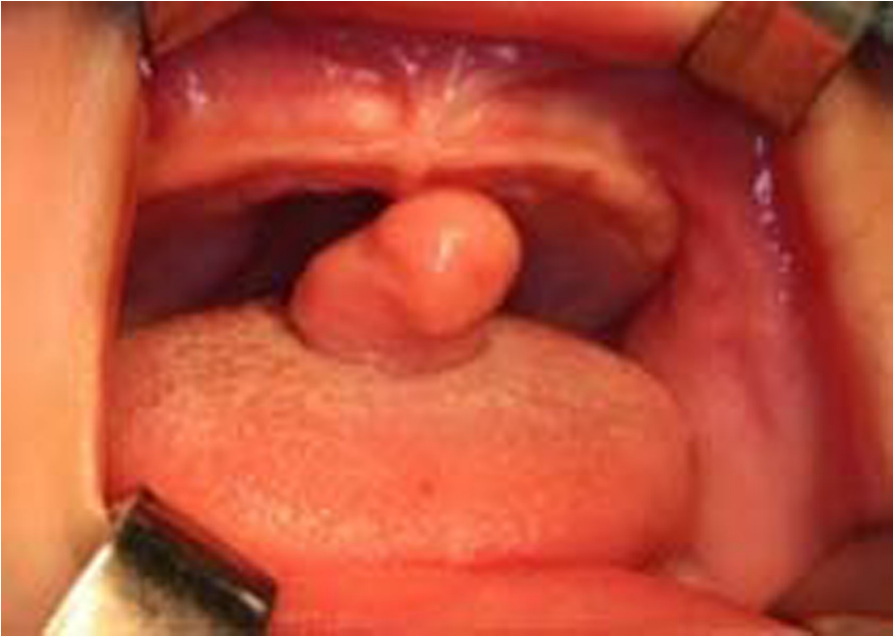
Image of the voluminous cylindrical lesion (phalic) on the dorsum of the tongue, erect and dislocated to the anterior portion of the oral cavity.

A new exam was set and when the computer images were obtained – for which the patient was sedated under spontaneous breathing – a pediculated mass with a cylindrical body could be seen (phallic). It was located longitudinally on the dorsum of the tongue, with its base placed in the median furrow, at an almost equal distance between the apex of the tongue and the foramen cecum. The lesion was mobile to any direction, composed of a soft consistency, yielding to traction and with color and texture similar to those of the oral mucous membranes (Figure 1).

**Figure 2:**
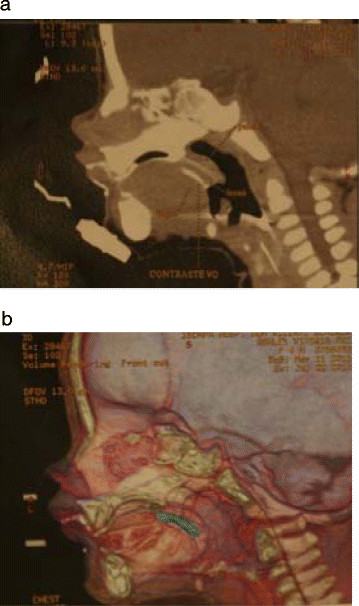
**a. Computed Tomographies viewed in sagital reconstruction and with topical oral contrast. **A hyperdense image is visible with its base on the dorsum of the tongue. **b.** The tridimensional reconstruction of the lesion, visible in blue color, demonstrates its relation to the skeletal structures**.**

Computed topography images were obtained. Through the use of a non ionic oral radiocontrastant a polipoid lesion was clearly distinguishable on the dorsum of the tongue, measuring 2,1cm x 0,8cm. The use of an oral radiocontrast allowed not only for the delimitation of the lesion's contours, but also, its dynamics, demonstrating its movement from the dorsum of the tongue to the oropharyngeal area, suspending the soft palate (Figure 2).

**Figure 3:**
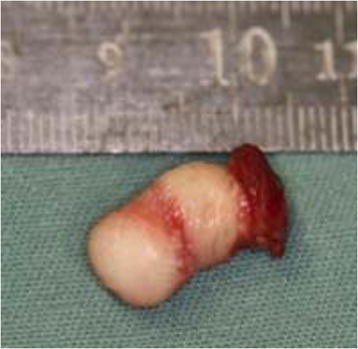
Image of the surgical specimen immediately after the resection.

Surgery was performed under general anesthetic, using nasotracheal intubation. A complete resection of the pedicle was performed, involving, in depth, the fibers of the superior longitudinal muscle of the tongue. After the hemostasis, the procedure was concluded with layer sutures to isolated points. The surgical specimen was stored in a 10% buffered formaldehyde solution and sent for anatomopathological analysis (Figure 3).

On the macroscopic examination a polypoid fragment of light brown tissue covered by a grayish and wrinkled mucous membrane was observed, measuring 0,8 x 0,9 x 0,8 cm. At the sections, in turn, it shows a bright light brown color.

**Figure 4:**
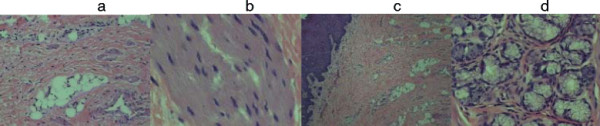
**Set of histological sections, demonstrating the existence of connective and adipose tissues, striated muscle, squamous mucous membrane, besides the salivary glands.** The lesion is diagnosed as Hairy Polyp, containing mesodermal and ectodermal layers. **a**. Connective and adipose tissues; **b**. Striated muscle; **c**. Squamous mucous membrane and connective tissue; **d**. Salivary gland.

The microscopic examination revealed a polipoid lesion lined with keratinizing squamous epithelium, containing stroma, besides connective and adipose tissue, smooth and striated muscle and salivary glands (Figure 4- a,b,c,d).

The postoperative period evolved without complications. In the five-month follow up there wasn’t any recurrence and the patient, therefore, was considered free from the lesion.

## Discussion

In spite of its uncommonness, the Hairy Polyp is recognized as a significant pathology that affects both naso and oropharynx. It’s a congenital tumor quite usual for those areas [6]. However, there are no cases of malignancy reported so far [10].

Displaying an approximate incidence of 1 per 40.000 births, most of the cases are considered isolated. According to Burns, Axon and Pahade [1], the Hairy Polyp is an abnormality of the development, and a common association with such malformation is related to the first and second branchial arches or to the nasopharyngeal membrane. For that reason, it could be linked to systemic alterations, for instance, the Oral-Facial-Digital Syndrome or Dysostosis [11], being essential a diagnostic investigation.

Carranza [2] regards the term *Hairy Polyp* as accurate and explanatory, for the process is considered as a developmental malformation. Its histology, though variable, is typical, and likely to be distinguished from other lesions that may affect the patient, dissociating itself from malign precesses. On the contrary to the epignathus, teratomas and trigeminal teratoids, the Hairy Polyp doesn’t have the endodermal layer in its origin [1].

The emergence of this lesion on the tongue frequently happens with children, up to 2 years old. In spite of appearing in different places, the Hairy Polyp is more often seen in the anterior area of the dorsum [11]. For Carranza [2], the obstruction of the upper airways can be reckoned as a sign of big lesions. Thus, it must be considered that, the large volume of the lesion reported in this case, besides compromising the vital functions, also hindered the conduction of more conclusive intraoral examinations.

Among possible imaging examination methods, there are ultrasonographies, radiographies and magnetic resonance. The advantage of computed tomography is its fast acquisition time, which facilitates the sedation. In spite of that, the use of ionizing radiation is a factor that must always be kept to a minimum necessary in these patients. The use of iodated oral radiocontrastant, in a reduced amount, dripped on the tongue’s surface, aggregated the advantages of lessening the time of sedation and exposing the area to viewing with a much higher resolution. In addition, it permitted the prompt comprehension of the extension and mobility of the lesion, which used to vary between distal and medial positions, constantly adapting itself to the oral functions.

The subsequently possibility of multiplanar and tridimensional reconstruction adds great academic value to the acknowledgment and comprehension of this pathology and its anatomical associations. In this case, it’s concluded that the whole outline of the process allowed the cylindrical mass, when positioned on the anterior part of the tongue, took on the functions of a pacifier. At a slight swallowing provoked by the contrast used, the lesion made a motion towards the opposite direction. Such a dislodgment, activated upon swallowing, creates favorable conditions for breathing and ingestion difficulties.

It’s possible that suction, when the lesion is moved anteriorly, might have sped up the growth of the lesion, justifying the complaints and the urgency in the intervention. With its body getting more and more voluminous, it was clear that there was a constant mechanical obstruction of the oropharynx.

The stimuli reflexes of coughing and liquid regurgitation, added to difficulties of the reflex between swallowing and breathing, hardened the patient’s feeding and led her into an increased irritability, expressed through crying. The presence of vomiting couldn’t be confirmed. The apprehension of this aspect at the location of the lesion equally guided the choices of orotracheal intubation and general anesthetic.

## Conclusion

This pathology deserves special attention since its existence, by obstructing the upper airways, compromises vital functions such as swallowing and breathing. Due to clinical similarities to other kinds of lesions, its recognition at the oral routine examinations of the child may sign to systemic diseases. The referred case brings out the Hairy Polyp as a process susceptible to fast development, making vital functions vulnerable, mainly with newborns. Therefore, it’s essential that the clinical diagnosis be accurate, so that the surgical ablative local treatment can be done, and the final anatomopathologic can be substantiated. The use of computed tomography with an oral radiocontrastant may not only aid in diagnostics, but also clarify the dynamics of the tumor.

The postoperative follow-up is key, not only to check that the healing happens without recurrence, but also to watch the functional response of the tongue in a developing and growing patient.

## Consent

Written informed consent was obtained from the patient for publication of this report and any accompanying images.

## Competing interests

The authors declare that they have no competing interests.

## Authors’ contributions

EP: surgery performed and documented the case. Drafted the manuscript. MBB: performed the histopathological analysis. BH: exams performed the analysis of the image. DP: performed surgery. HVA: performed the surgery and review of literature. Drafted the manuscript. MAM: conducted a review of literature. Drafted the manuscript. JHS: anesthesia carried out in obtaining the CT scan and at surgery. All authors read and approved the final manuscript.
